# Prior employment as a welder associated with the development of chronic myeloid leukaemia.

**DOI:** 10.1038/bjc.1988.173

**Published:** 1988-07

**Authors:** S. Preston-Martin, J. M. Peters

**Affiliations:** Department of Preventive Medicine, University of Southern California School of Medicine, Los Angeles 90033.


					
B C ( 5 5  The Macmillan Press Ltd., 1988

SHORT COMMUNICATION

Prior employment as a welder associated with the development of chronic
myeloid leukaemia

S. Preston-Martin & J.M. Peters

Department of Preventive Medicine, University of Southern California School of Medicine, 2025 Zonal Avenue, Los Angeles,
California 90033, USA.

Welding is an occupation that involves a complex set of
exposures to the worker which varies depending on the
specific welding technology and on the materials used. There
are about 20 major welding technologies which are applied
to ten major classes of materials, and thousands of different
sets of occupational exposures can occur (Stern, 1981).
Studies suggest that welders have decreased lung function
(Oxhoj et al., 1979) and are more likely to die from
malignant or non-malignant respiratory disease (Beaumont
& Weiss, 1980, 1981; Polednak, 1981).

We report a strong association between chronic myeloid
leukaemia (CML) and prior employment as a welder. In
addition, we present some details relating to the welding job
held by study subjects in an attempt to identify factors which
might relate to the development of CML. Our findings
support recent reports of an increase in myeloid leukaemia
among welders (Stern et al., 1986; Savitz & Calle, 1987).

The patients were Los Angeles County residents, aged 20
to 69 years, with histologically confirmed CML first
diagnosed from April 1, 1979 through June 30, 1985. The
patients were identified by the Los Angeles County Cancer
Surveillance Program (CSP), the population-based cancer
r egistry for Los Angeles County. As the questionnaire
sought detailed information on past occupational exposures
and on radiographic examinations during the 20 years prior
to leukaemia diagnosis, we restricted the study to living
patients.

The CSP identified 229 eligible cases. Their attending
physicians granted permission to contact 206 (90%) of these
patients. We were unable to locate 41 patients and 28
patients refused to be interviewed. We therefore obtained
completed questionnaires on 137 (83% of the 165 patients
contacted about the study or 60% of all eligible cases).

We sought an individually matched neighbourhood
control for each of 137 cases. Each control matched the case
on sex, race (black or white) and birth year (within 5 years).
Both cases and controls had to be able to be interviewed in
English. Matching on neighbourhood of residence resulted in
a close match on socioeconomic status (SES) as determined
by occupation and level of education (Hollingshead, 1957).

To find the neighbourhood controls, we used a procedure
that defines a sequence of houses on specified neighbour-
hood blocks and our goal was to identify the first matching
resident in the sequence and to get his telephone number so
that he could be contacted by the interviewer. If no one was
home at the time of the visit, we left an explanatory letter
and made a follow-up visit after several days. In 102 (74%)
instances, the first appropriate person agreed to cooperate.
When the first matched control refused to participate, the
next in the sequence was sought. In all, 136 matched
neighbourhood controls were found and questionnaires
completed.

All interviews were conducted by telephone by four

Correspondence: S. Preston-Martin

Received 8 December 1987; and in revised form, 4 January 1988.

interviewers; both members of a matched pair were inter-
viewed by the same interviewer. Because we explained to the
subjects how we obtained their names, the interviewer was
not blind as to each subject's status. Information was
obtained by use of a standard questionnaire which included
a verbatim script. The major portion of the interview asked
about diagnostic radiography, radiation treatment and use of
certain drugs. In addition, study subjects were asked if they
ever worked in eleven specific occupations or industries;
these eleven were chosen because of their suggested possible
relationship to leukaemia risk.

Six pairs were excluded from these analyses because either
the case (3) or the control (3) had a history of radiation
treatment to the trunk. None of the 130 cases used in this
analysis had a history of cancer chemotherapy. The matched
pair design was maintained for the analysis. A crude odds
ratio (OR) was calculated for the factor 'prior employment
as a welder'. This factor was also included in a conditional
logistic regression analysis and an OR, adjusted for all other
factors, was calculated. All statistical methods used are
described in detail in Breslow & Day (1980).

Of the 130 cases and 130 controls included in our analysis,
22 cases and four controls had worked as a welder at some
time prior to the year of diagnosis of the case. Similar
numbers of cases and controls had worked in each of the
other ten occupations or industries queried. Three pairs were
concordant for having worked as a welder. Among the
remaining 20 discordant pairs, only the case was exposed in
19 and only the control was exposed in one. This
distribution yields a crude matched OR of 19 which is
statistically significant at the P<0.01 level. In a conditional
logistic regression analysis, the OR for welding increased to
25.4 after adjustment for the effect of other variables (rad
exposure to the active bone marrow from diagnostic radio-
graphy; having ever lived on a farm) with a 95% confidence
interval of 2.78-232.54 (Table I). When the analysis was
restricted to only those subjects whose job title included the
word 'welder' or 'welding', the crude matched RR was
infinity (15 cases and 0 controls; P<0.01).

Table I also lists diagnostic information (year and age at
diagnosis) and some details of the welding jobs held (years
employed as a welder; type of industry; job title; details of
welding process and materials; and exposures explicitly men-
tioned during the interview). This information is listed first
for those 19 pairs where only the case was exposed, then for
the three concordant pairs (subjects 20-25) and finally for
the discordant pair where only the control was exposed
(subject 26). All subjects who were welders were male except
two cases (numbers 10 and 13) who worked as welders
during World War II and one control (number 26) who
worked as a welder in the 1960s.

Nine of the cases and none of the controls had worked at
welding jobs for five or more years. Fourteen cases used arc
or heli-arc welding. The job exposures most commonly
mentioned included welding fumes, smoke or gases (13 cases;
3 controls); carbon tetrachloride (2 cases; 1 control);
unspecified solvents (4 cases; 0 controls); and X-rays used to
examine the weld (2 cases; 0 controls). Other exposures

Br. J. Cancer (I 988), 58, 105-108

106  S. PRESTON-MARTIN & J.M. PETERS

cq                uA

-o
= 0

V-C

U-':

_  0

ci s '
O.

4 -4 .

_A 04

V "

0
0.

C)

-

0
,,
X~~~~~~~~r
*m~~~~

u: B

C  CA

co)  0

.. ..

) CA

Cd  _  a4

00 Q

C-

U

t Q ooU  ^C

6

*_,                ~~~~0O

6-     6 ,

.4)

w          A
o
o }-4

0
00

N
-41

0
00

0%-
0%

1-:

0 r4O

t- r t
en _% 0%

0~~~~~0

CA. d C '

U ~~~0 U -~~o c

CA ~~~~~~~~~~~~~~~C

0 0 ~

CA3   C A  -

0400~ ~~

104
CAd

Uc *

W '0

0

CA      00Q

'U'^ . C

D0~'      '0

_  q

0
0.

-'~~C      C) Y'  Uo
I-       Y   CS  ,~  -
ooO ~ ~  O*  C)  .  -,  U

C)0  C)'.-

4 ...  00
C)

00

I

ON
0%
0%1

t-
r-
ON
-%

00
CS

1-

'In
0%

0   110

cEj -

I

. I

cq   I _4  _
lt .^e 1t  t-
_a5 - ONC

1-     -    00 -     en     0%       Cf     00        0
WI)        WNI)'.0 v  t     el       .1      -        . 2

o        o   -- -      (N
00     00    00 00 00  00
O%           al l%0% O   ON

oo  o    o oo o   oy 1-

00          00       00    00   00    00
0%         0%       0%    0%    0%    0%

e- ci i ri   N.6     &-        o6      or~ 6          C4

C.)
Uj

S:0 C

U n0

NsC
0% c

('I -ts

144 %)
16. r
:2    E

C'n Q

Q *4
Q. x
x

44

.4

11 ?3

So .16.
-m 14)

?u I

5t

". -,,s
Q,

.4 ?3
.- cn
M   rn

4Q %)
qu Q
q Q

Pl.

Q.

wi
00

0%

4)
U
00
0

B
0
U

00

U

0
0

C)
0

,0
C)

0
U

C._

C)
0Y

0_

0

0_

U)

.z

C'0
c'0

4-a
0:
C)

a

cq
113
O

CA)

ro

03'd

,CA

U

to

Q Q.
QS8

Il4

Q
CA

6.-
10

u N

U

Q.

ot~~~~~~~~~~r

44

CO0
0Q

Uz
U-

OCA,
0,  1
00 I

x

*0~-tk

WELDING AND CHRONIC MYELOID LEUKAEMIA  107

0

0   C C.

CU     4)

U4 0
o   d

o4e  )

00,

4) ,  4-  E -
?b? C)   '

o  o

0      0~~~~~0

00

C.0 ~ ~ ~ '

C U '- 0   )-

Cd  0.) ~ ~

,0 '0 C  U,
CU  0 '   )   x , ;

CU a

C)
4)
0

4) E C)4?

>

0                    4) o B

~~  -4   0~ ~

u0   0                  -

U    ,  * 'Q  4)  U

.W00                    C 3U

w~~~

;Y      0

0                                                        0  o

4) o'-4                                                v

0                                                        0o 00

~~~   0                        4 )       - 4~~~~~~~~0 e

000 3                                                     0 w  _

c R  ?        3?   r        ?     ;        ? t~~~~~~~~~~~4
3,                                            00       U ,.3  n

4)

Cd  ~ ~ ~ ~~) Cu  (4     Cu
-    0  2 'w - 8  CU    )  4

6   ci

0   C   * 4u 0 ,

, C.)  C.)  CU
' 0w   )  ..>     4 C  -   . b   .

CUC),o'0~~~  C)C),1            Cu

'c&0 0   S 0   0  0  4) ) 4 0 ~  0

0%  I  0t   I  00  ' W

'N-4 C., ~-W7 ~-1(O

1- :>r isX  I :Xo

_ '~ 0 ' - _   __

0   %. (7 0 %' en C'

-4

%n  I  oo

tn  t

_ ,ON
-o4: -

U)
00

0%~

-0

1

W)  'O Rt   vo                     en   C m

0

N    0%   -   '0   0l   0      0      o0

*uu
16.

'r~~ ~~.0 ~~  ~~.0  'r4-~~~~fo  C.0 '0r

Q

F oo Oo o o -~4

: 0

;0

0    ;" i

.   -

I     L. -

-B-B.

o1 w-oo-1
1-o =. U)-

oy. o~ ofi o~

_      kn_

00    00

00

0   --
-      0

cl

in

0%

ON.

1

Cq

r-          ONa

N t          0 %

000

0-        +.0

a ll  Cl- -. C

0

U

*CE
CO)

Q

MW,  o o

Q N  :

4   i  6

0

o

00
0

0
>
'00

4)
C), *
ce

CA

oa0

0._

cU

o U,

>

s4)

'00
CA

E 00

u0
0
wD

o v

4)._

0

CU

4.)
C)4

orA
CU4

> 00

rA

U,0

CU Q

D

0 O
.D--

CU;
*4.0

4)*_
4u) 4.-
4)C^

o0 r

108  S. PRESTON-MARTIN & J.M. PETERS

mentioned included paints, cooling oils, epoxides, metal dust,
fibre glass dust, asbestos, and exhaust fumes. Poor ventil-
ation and inconsistent use of protective equipment were also
mentioned.

Among cases, the interval between year of first welding
job and year of diagnosis ranged from two to 44 years. Four
cases first worked as welders 40 or more years prior to
diagnosis; five 30-39 years prior; four 20-29 years prior; six
10-19 years prior and only three first worked as welders
within ten years of diagnosis. Only six of the 22 cases were
still working as welders at the time of their CML diagnosis.

Subjects who had ever worked as a welder were asked the
years worked; the name of the company they worked for and
what the company made or did; their job title and what they
did on the job. No explicit questions were asked about the
welding process or materials used. We also did not ask
specifically the number of hours per day or week spent
welding, about ventilation of the work site or about the use
of protective clothing or equipment, although some subjects
volunteered this information. All these factors influence the
dose of various exposures incurred by welders. Our study
therefore, has limited information on possible common
welding exposures experienced by these 22 CML cases and
on the probable level of such exposures.

Most cases did arc or heli-arc welding, technologies that
use electrodes coated with any of over a hundred different
compounds (Stern, 1981). Welding with coated electrodes
has been associated with a higher cancer risk than welding
with other processes (Becker et al., 1985). Welders can be
cxposed to various established or suggested leukaemogenic
aigents including solvents used to clean the surfaces welded
together, X-rays used to check the welds, and heightened
exposure to electro-magnetic fields.

We considered several possible sources of bias or

confounding which might explain this finding. Because
working as a welder is likely to be related to SES, we
compared the SES distribution of the 60% of all eligible
cases included in the study to that of the 40% not included
and found that the distributions were similar when SES was
determined by census tract of residence. Interviewers were
not blind as to the case or control status of each subject;
however, the questionnaire used as a verbatim script and
standard probes were used in a prescribed manner. Subjects
were told that the study aimed to get information that might
tell something about the causes of certain diseases. In
addition, they could tell that the focus of the questionnaire
was on radiography. Of the eleven specific occupations and
industries queried, only welding appeared to be associated
with CML. Because cases and controls were matched on sex,
year of birth and SES there was no need to control for these
potentially confounding factors in the analysis. Also, the
strength of the welding association was not weakened when
we adjusted for other risk factors in a multivariate analysis.

The striking frequency of welding occupations in the cases
suggests a causal connection between welding exposures (or
something   very  closely  associated)  and  subsequent
development of CML. The level of detail of information
collected in this study does not shed light on whether gases,
fumes or radiation (whether ionizing or non-ionizing, such as
clectric and/or magnetic fields) are responsible. Future
studies should acquire more detailed information on specific
exposures and welding tasks and should measure exposures
to gases, fumes and radiation where possible.

This work was supported by grant SIG-2 from the American
Cancer Society.

The authors thank Dr D. Thomas, Dr M. Yu, A. Chang and K.
Arakawa for technical assistance.

References

BEILAUMONT, J.M. & WEISS, N.S. (1980). Mortaility of welders,

shipfitters, and other metal workers in boilermakers local no.
104, AFL-CIO. Am. J. Epidemiol., 112, 775.

IlAUMONT, J.J. & WEISS, N.S. (1981). Lung cancer among welders.

J. Occup. Med., 23, 839.

BFCKER, N., CLAUDE, J. & FRENTZEL-BEYME, R. (1985). Cancer

risk of arc welders exposed to fumes containing chromium and
nickel. Scand. J. Work. Environ. Health, 11, 75.

BRESLOW, N.E. & DAY, N.E. (1980). Statistical methods in cancer

research. In The analysis of case control studies, (ed) p. 211.
IARC: Lyon.

HOLLINGSHEAD, A.B. (1957). Two Factor Index of Social Position.

New Haven, Conn.

0XII;O.. H.. 1HAKE. B.. WlIELL. H. & WILHELMSEN, L. (1979). Effects

of electric arc welding on ventilatory lung function. Arch.
Environ. Health, 34, 211.

POLEDNAK, A.P. (1981). Mortality among welders, including a

group exposed to nickel oxides. Arch. Environ. Health, 36, 235.
SAVITZ, D.A. & CALLE, E.E. (1987). Leukaemia and occupationil

exposure to electromagnetic fields: Review of epidemiologic
surveys. J. Occup. Med., 29, 47.

STERN, F.B., WAXWEILER, R.A., BEAUMONT, J.J. & 7 others (1986).

A case-control study of leukaemia at a naval nuclear shipyardl.
Am. J. Epidemiol., 123, 980.

STERN, R.M. (1981). Process-dependent risk of delayed health effects

for welders. Environ. Health Persp., 41, 235.

				


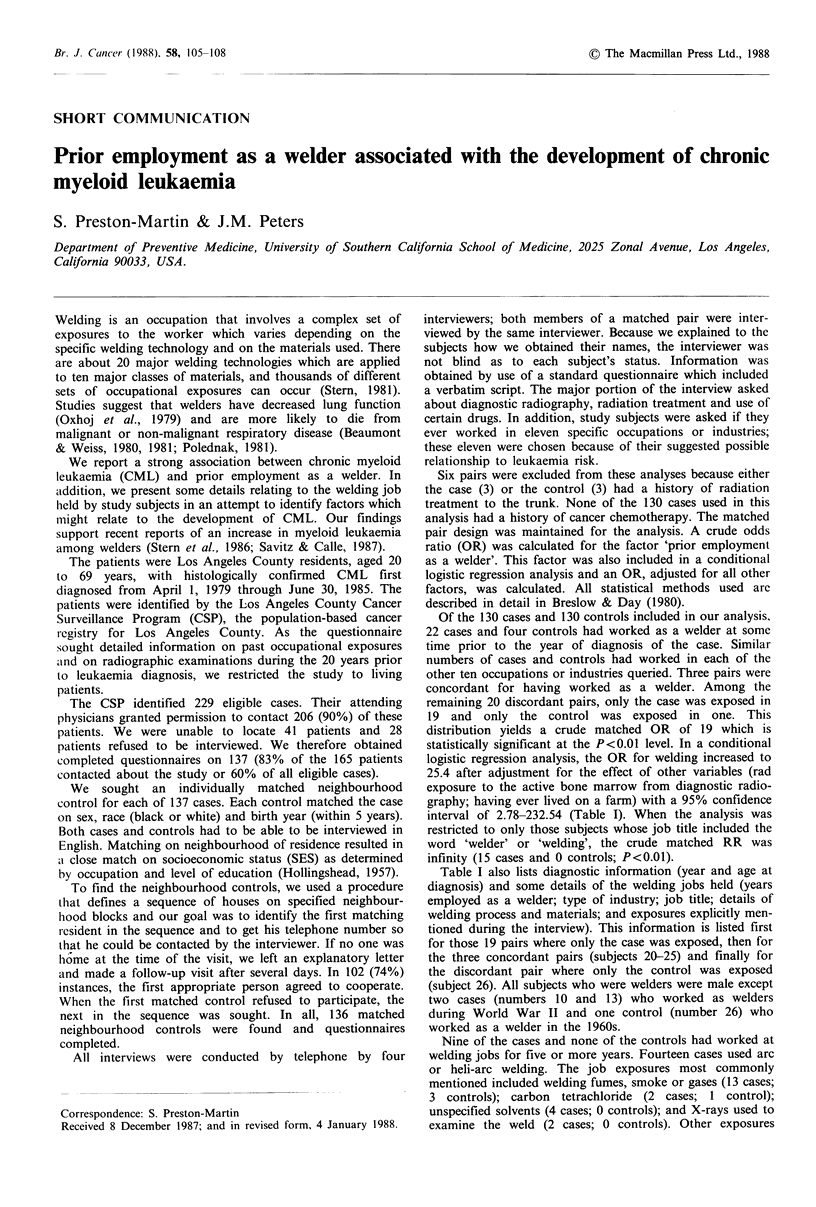

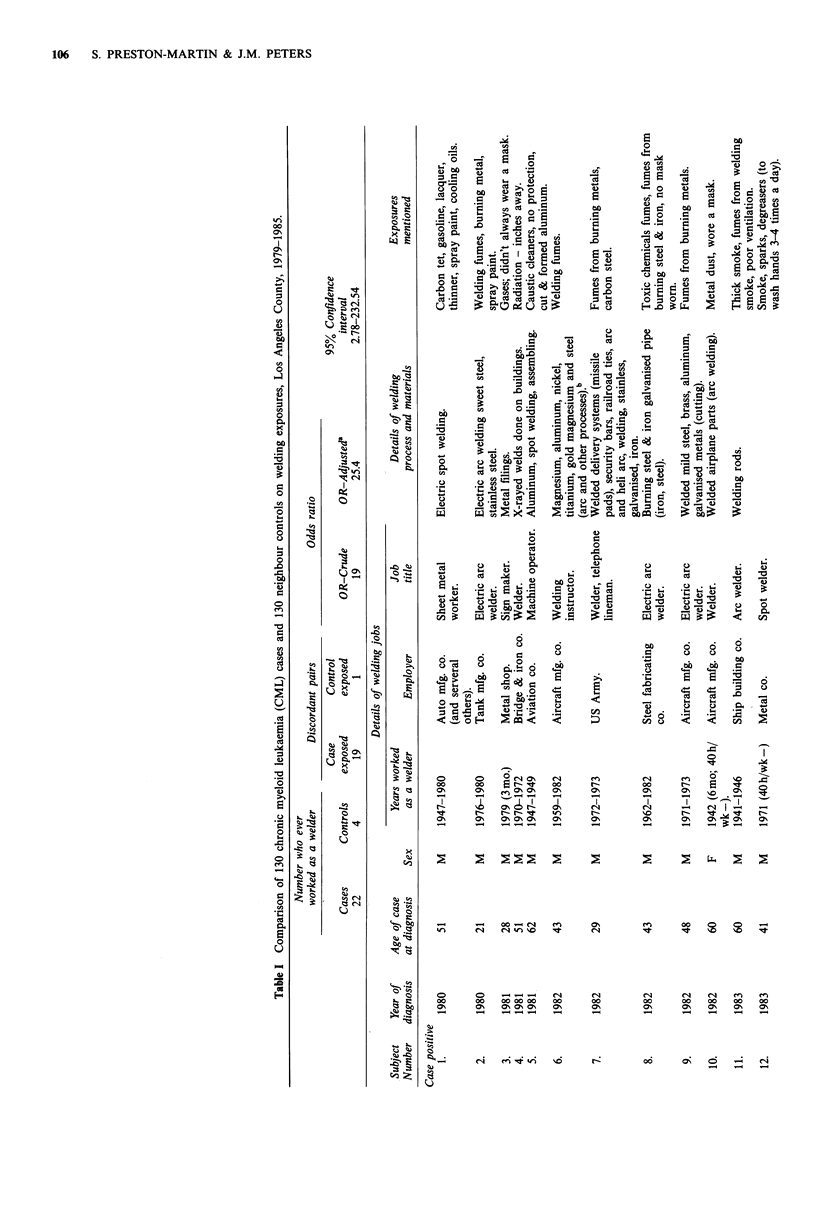

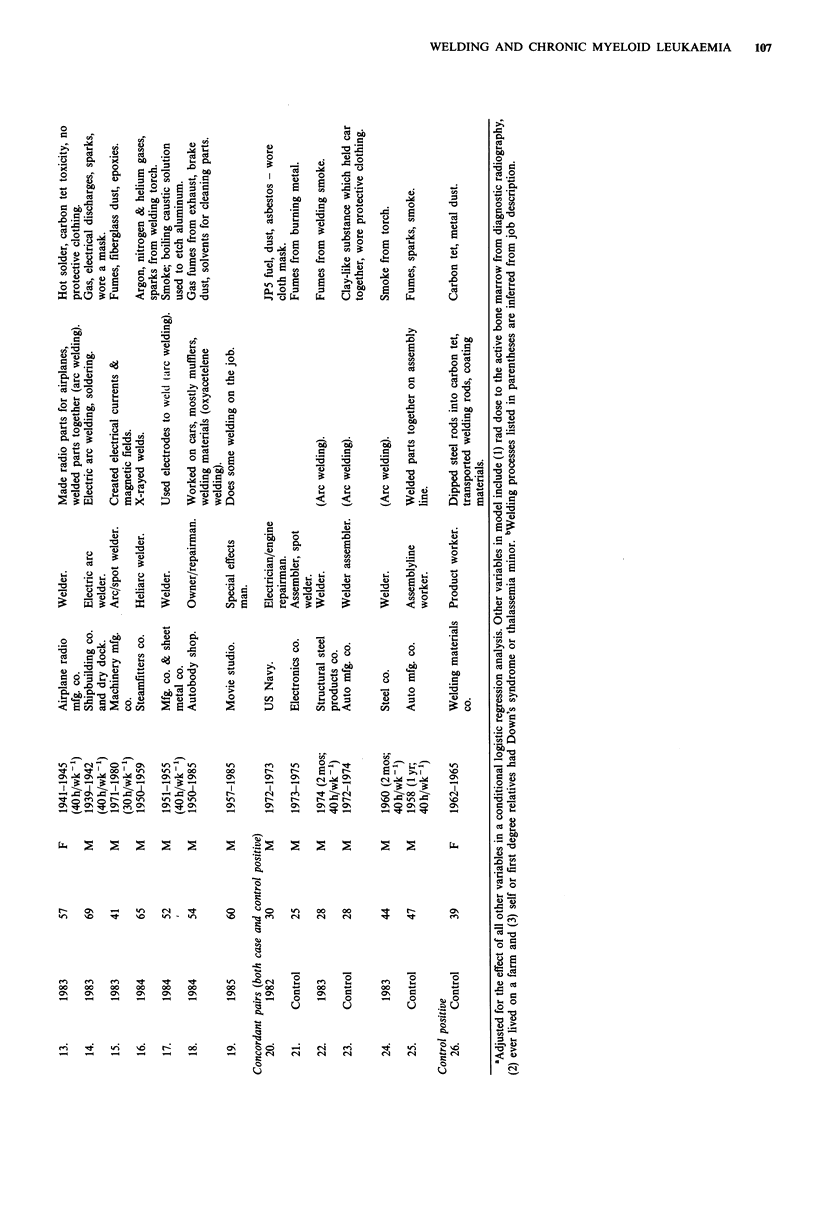

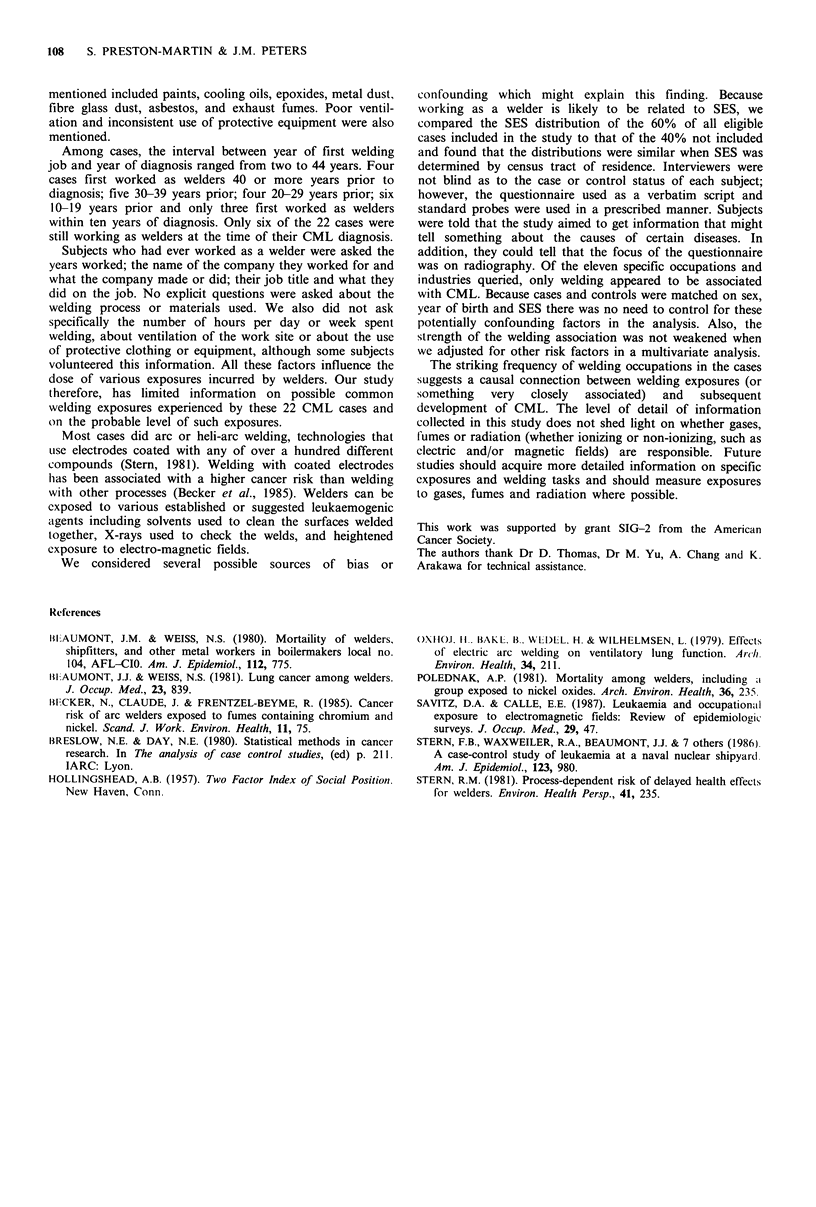

